# Practical Chemistry for Students and Physicians—Chapter Fourth

**Published:** 1883-10

**Authors:** 


### Editorial.

### PRACTICAL CHEMISTRY FOR STUDENTS AND PHYSICIANS—CHAPTER FOURTH.

(Continued from September Register, Page 754.)

*Chlorine and Its Group.*—The next elementary gas is chlorine, from the Greek *cloros*, green, referring to its characteristic, yellowish-green color, the most important member of a very interesting group in chemistry. The other members are bromine, iodine and fluorine, each having the terminal *ine*, to indicate their close relation. They are so much alike in their chemical properties that what may be written of chlorine will be nearly applicable to each of the others as well.

These elements abound in sea-water, and for that reason have been called the *halogen group*, from *als*, the sea.

*History of Chlorine.*—This gas was discovered by Scheele, a celebrated German chemist, in 1774, the same year with the discovery of oxygen by Priestley. It was not, however, till nearly thirty years later, that its elementary character was demonstrated by Davy, of England, and when it received from him the name of chlorine.

Scheele, saturated with the old theory of flogiston, like Priestley when he gave name to oxygen, had called it deflogisticated muriatic acid. The symbol of chlorine is Cl.

*Occurrence of Chlorine.*—Every liter of ordinary sea-water contains five liters of chlorine gas. It is only found in combination, as with sodium, magnesium, potassium and calsium. In union with sodium, it is exceedingly abundant in vast beds of rock salt. Sodium chloride is the principal source of the gas for all practical purposes. On a smaller scale it may be derived from the action of hydrogen chloride, HCl, upon manganese dioxide—$MnO_2$.

*Apparatus for Generating and Collecting Chlorine.*—This is the same as that used in the collection of oxygen, except that the chlorine, being considerably heavier than air, may be procured by displacement—that is, in a jar which is neither filled with water nor inverted over it. The air in the jar is gradually displaced as the chlorine enters, until it is full of the gas.

It could be collected over water, as hydrogen and oxygen, but with large waste of the chlorine, for one gallon of water will dissolve three gallons of the chlorine.

An ordinary wide-mouthed jar used for preserves, having a pasteboard cover, pierced by an arm of the delivery tube, and reaching nearly to the bottom of the jar, is most suitable for the purpose. The cover is to prevent the too rapid diffusion of the gas into the air of the room. Chlorine is so irritating to the air-passages that it can only be generated in a close room, in small quantities, and cautiously.

*Method of Preparation.*—Put in a test-tube about 120 grains of common salt powdered, and the same amount of $MnO_2$, well mixed together, and cover them with water. On adding a little strong sulphuric acid, the gas will be evolved at once. Gentle heat increases the evolution. The characteristic odor of the gas, and the yellowish-green color of the jar or test-tube will sufficiently indicate its presence.

If the jar or test-tube be first filled with water, the solution of the gas in the liquid will form the official Aqua Chlorinii U. S. P., a valuable reagent of the laboratory, to which we shall again recur. For experimental purposes, chlorine may be obtained from hydrochloric acid, HCl, and manganese dioxide, $MnO_2$. The sulphuric acid was used in the above process only to supply from the salt hydrochloric acid, which in its turn, acted upon by the manganese, gave free chlorine.

*Process by Hydrochloric Acid.*—Place in a test-tube or flask, one part of the manganese dioxide, and four of the acid. Gentle heat will cause the gas to be evolved at once. This is the method of the Pharmacopæias for obtaining chlorine, even on a large scale. The first process is detailed as follows, by high authority :

| | | | |
|---|---|---|---|
| Take | Common salt in powder | 36 | parts. |
| | Manganese dioxide | 30 | " |
| | Strong sulphuric acid | 50 | " |
| | Water | 42 | " |

First mix the acid with the water in a flask, and when cool, add the salt and manganese previously well mixed together. The gas comes over at once, and when it ceases, heat gently eight grams, or about 120 grains of manganese dioxide, and 20 grams, or about 300 grains of commercial hydrochloric acid, are required to yield one liter cf chlorine gas.

*Physical Properties of Chlorine Gas.*—This is the only one of the elementary gases that has color, taste and odor. Its odor is suffocating, but is said to be suggestive of the sea. The taste is astringent. No animal can breathe it; it first irritates and then inflames the air passages. In water its solubility is such, that at 12.2 F. one volume of water will take up three of the gas. The specific gravity of chlorine is 2.46, nearly two and a half times heavier than air. Its density is 35 5, and molecular volume 2. One liter weighs 3.17 grams.

*Chemical Properties of Chlorine.*—The two distinctive properties of chlorine are first, its great attraction for hydrogen, and second, its valuable bleaching powers, the one depending upon the other. Its chemism, or chemical activity, is quite intense; excepting oxygen, nitrogen and carbon, it combines directly with all the elements, especially the metals. Hence chlorine does not burn in air at the highest temperatures; thc chloride of carbon is the rarest of substances, and nitrogen chloride one of the most unstable and dangerously explosive compounds known to chemists.

The atomic weight of chlorine is 35.5; its quantivalence I, III, V, VII.

This gas is a powerful deodorizer and disinfectant for the same reason that it is a powerful bleacher; viz: because of its great affinity for hydrogen. In no sense is chlorine an antiseptic. The student should have a clear perception of the difference between a disinfectant and an antiseptic. Yet there is, from the nature of the subject, much confusion in the popular mind in regard to a practical difference between them, and no little looseness of expression, even in the books. One standard author* says: "Chlorine readily decomposes offensive effluvia; it is one of the most powerful of the deodorizers. It also decomposes putrid and infectious matter; it is one of the best of *disinfectants*. Antiseptics are subjects which *prevent* putrefaction." Another,† of equal authority, writes: "In this respect—by destroying the vitality or otherwise preventing the development and propagation of microphites—*antiseptics* are distinguished from *disinfectants*, the action of the latter being directed only towards the exciting causes and offensive or deleterious products of a class of changes which are themselves more comprehensive than those implied by the term putrefaction." He then goes on to enumerate special antiseptics, and includes chlorine among the chief of them.

The difficulty is that some of the best disinfectants and deodorants are, also, among the most powerful septics known to chemistry, notable examples of which are chlorine and charcoal, because acting directly on the noxious products of putrefaction they promote combinations which are no longer susceptible of decomposition by the presence of living organisms—the micrococci. Neither chlorine nor charcoal exert any direct effect upon contagium; they only destroy the pabulum and break up the breeding places of contagion.

Chlorine is a disinfectant and deodorant, because the new proximate compounds formed by its agency, such as water; phosphoric and sulphurous acids are innocuous and inoffensive, or powerfully promotive themselves, in their turn, of similar innocuous compounds.

This valuable action of chlorine is wholly due to its affinity for hydrogen. For the same reason it exerts its useful powers as a *bleaching* agent, combining with the hydrogen of the water in the coloring matter of all vegetable substances, and setting oxygen free, the colors are per-

---

*Attfield.

†Prof. Redwood in Quain's Dictionary.

manently discharged. Moisture is essential to the bleaching process of chlorine.

*Chlorine and Hydrogen.*—With all the strong attraction of chlorine for hydrogen, there is but one known compound of their union, hydrogen chloride, or as it is commonly called, hydrochloric acid.

*History of Hydrogen Chloride*—This gaseous acid is said to have been known to the ancient Egyptians; it was certainly known to the alchemists, who named it in their quaint language "spirit of salt." It received the name it bears in the old chemistry, "muriatic acid," from the alchemist Glauber, from the Latin *muria*, brine. It was first obtained in its purity, 1772, by Dr. Priestley. Nearly forty years later, Davy first showed its true composition.

*Preparation of HCl.*—Fill one-half of a strong jar with chlorine, and the other half with hydrogen, in the manner already described, and apply a flame to the open mouth. The two gases will instantly combine with explosion and form HCl, whose volume will occupy exactly the same space as the two gases before combining.

*Common Hydrochloric Acid.*—This body, or this Acidum Hydrochloricum of pharmacy, is simply a solution of the acid gas in water, forming the liquid acid. The method of its formation with its chemical and physical characteristics will be given in the proper place.

*Nitrogen and its Name.*—The next, and last of the elementary gases is nitrogen, a name analogous to those of hydrogen and oxygen, having the same terminal syllable, *ene*, but without any group relationship, as would at first appear. It comes from the Greek, *nitron* and *gennao*, niter former, since nitrogen is an essential ingredient of niter, $KNO_3$.

*History of Nitrogen.*—Nitrogen was discovered by Rutherford, just two years before the discovery of oxygen by Dr. Priestley, in 1774. The method by which he proved the existence of this new gas is interesting. Having collected a volume of air, which had been breathed by an animal, he caused it to pass through a solution of lime in water. There emerged a gas which was neither respirable nor a supporter of combustion.

What is the explanation of this? Common air passed thus through lime-water suffers no change except to be made purer and more capable of sustaining both respiration and combustion. It must be remembered that respired air is composed chiefly of carbonic dioxide, $CO_2$ and nitrogen gas, and that lime has a powerful attraction for the carbonic dioxide, forming with it in the water solid particles of chalk or $Ca\ CO_3$. Hence the result.

After Rutherford's discovery, Lavoisier proved that this new gas made up four-fifths of our atmosphere, and called it azote, from *a* and *zoe,* without life. Chaptal named it nitrogen.

*How Nitrogen Occurs.*—It exists in vast quantities in the atmosphere in a free state. It is an essential constituent of vegetable and animal substances, and in combination with the metals, is exceedingly abundant.

*The Preparation of Nitrogen.*—If it be recollected that the air is composed chiefly of the two gases, nitrogen and oxygen, four-fifths of the first and one-fifth of the latter, and that oxygen is easily burned up by many substances, such as phosphorous, hydrogen, sulphur, etc., it will be easily understood how nitrogen is usually obtained.

Place a piece of dry phosphorus, the size of a large buck-shot, on a small plate or dish and float it upon water in a shallow dish. Ignite the phosphorus by means of a hot metallic rod and invert over it a jar or wide-mouthed bottle of about half pint capacity. The combustion of the phosphorus by the oxygen of the air confined in the jar, quickly fills it with phosphoric pentoxide, $P_2\ O_5$ a white floculent solid, which is slowly absorbed by the water in which the mouth of the jar is immersed, leaving nearly pure nitrogen with about one-fifth of the capacity of the jar occupied by water. The water ascended to take the place of the consumed oxygen.

*Physical Properties of Nitrogen.*—This gas, again, like hydrogen and oxygen, is colorless, odorless and tasteless. Its specific gravity being 0.71, it is lighter than air and with the other elementary gases, is now, no longer irreducible. One hundred liters of water will dissolve about two and a half liters of nitrogen.

*Chemical Properties of Nitrogen.*—Its chief chemical characteristic is that it has no positive properties of its own. It is not combustible, nor is it a supporter of combustion.

Its chemical properties?

